# Relationship between amyloid and tau levels and its impact on tau spreading

**DOI:** 10.1007/s00259-021-05191-9

**Published:** 2021-01-26

**Authors:** Vincent Doré, Natasha Krishnadas, Pierrick Bourgeat, Kun Huang, Shenpeng Li, Samantha Burnham, Colin L. Masters, Jurgen Fripp, Victor L. Villemagne, Christopher C. Rowe

**Affiliations:** 1 Health and Biosecurity Flagship, The Australian eHealth Research Centre, Melbourne, Victoria Australia; 2 Department of Molecular Imaging & Therapy, Austin Health, LVL1 Harrold STOKES Block, 145 Studley Road, Heidelberg, Melbourne, Victoria 3084 Australia; 3 Health and Biosecurity Flagship, The Australian eHealth Research Centre, Brisbane, Queensland Australia; 4 The Florey Institute of Neuroscience and Mental Health, The University of Melbourne, Melbourne, Victoria Australia; 5 Department of Medicine, The University of Melbourne, Melbourne, Victoria Australia; 6 The Australian Dementia Network (ADNeT), Melbourne, Australia

**Keywords:** Tau imaging, Aβ-amyloid imaging, Alzheimer’s disease, Neurodegeneration, Positron emission tomography, Tauopathies

## Abstract

**Purpose:**

Previous studies have shown that Aβ-amyloid (Aβ) likely promotes tau to spread beyond the medial temporal lobe. However, the Aβ levels necessary for tau to spread in the neocortex is still unclear.

**Methods:**

Four hundred sixty-six participants underwent tau imaging with [18F]MK6420 and Aβ imaging with [^18^F]NAV4694. Aβ scans were quantified on the Centiloid (CL) scale with a cut-off of 25 CL for abnormal levels of Aβ (A+). Tau scans were quantified in three regions of interest (ROI) (mesial temporal (Me); temporoparietal neocortex (Te); and rest of neocortex (R)) and four mesial temporal region (entorhinal cortex, amygdala, hippocampus, and parahippocampus). Regional tau thresholds were established as the 95%ile of the cognitively unimpaired A- subjects. The prevalence of abnormal tau levels (T+) along the Centiloid continuum was determined.

**Results:**

The plots of prevalence of T+ show earlier and greater increase along the Centiloid continuum in the medial temporal area compared to neocortex. Prevalence of T+ was low but associated with Aβ level between 10 and 40 CL reaching 23% in Me, 15% in Te, and 11% in R. Between 40 and 70 CL, the prevalence of T+ subjects per CL increased fourfold faster and at 70 CL was 64% in Me, 51% in Te, and 37% in R. In cognitively unimpaired, there were no T+ in R below 50 CL. The highest prevalence of T+ were found in the entorhinal cortex, reaching 40% at 40 CL and 80% at 60 CL.

**Conclusion:**

Outside the entorhinal cortex, abnormal levels of cortical tau on PET are rarely found with Aβ below 40 CL. Above 40 CL prevalence of T+ accelerates in all areas. Moderate Aβ levels are required before abnormal neocortical tau becomes detectable.

**Supplementary Information:**

The online version contains supplementary material available at 10.1007/s00259-021-05191-9.

## Introduction

The two prominent neuropathological features of Alzheimer’s disease (AD) are extracellular aggregated Aβ-amyloid (Aβ) and intracellular aggregated tau in the form of neurofibrillary tangles. Post-mortem studies [1–3] suggest that these two pathologies occur in a different sequence and following different spatial patterns. Recent advances in position emission tomography (PET) allow for the in vivo quantification of these pathologies [4]. This offers a unique opportunity to better understand the chronology of appearance and the interaction of these two pathologies and this will contribute to unravelling the complexity of the disease and may enable more accurate diagnosis and prognosis of Alzheimer’s disease.

Aβ PET has been available for the last 15 years [5] and has been widely used and studied in research settings. Hence, the relationship between Aβ PET, CSF biomarkers [6], APOE genotype [7–9], brain atrophy [10, 11], plasma Aβ [12], and clinical phenotype [13, 14] has been thoroughly examined. However, tau imaging has been proven more challenging [15], due to the intracellular nature of tau [16]. The first selective tau tracer was only presented in 2011 [17], quickly followed by other first-generation tracers such as [^18^F]Flortaucipir (FTP) (aka AV1451, T807) and [^11^C]PBB3 [18].

A recent cross-sectional study demonstrated the interaction between Aβ and tau in a large cohort of clinically characterized individuals using [^18^F]FTP [19]. This study supports the hypothesis that Aβ triggers the spread of tauopathy into cortical regions and it is this spreading tauopathy is then responsible for cognitive impairment. The aim of the current study was to determine the interaction between Aβ and tau across the AD continuum using [^18^F]MK6240 and [^18^F]NAV4694 to establish the mean Aβ level associated with tau spread into the cortex.

## Method

### Participants

Four hundred and sixty-six participants were recruited from the Australian Imaging Biomarker and Lifestyle Study of Aging (AIBL). The full methodology for cohort recruitment and evaluation is detailed elsewhere [20]. All relevant institutional review boards approved the AIBL study, and written informed consent was obtained from all participants. Briefly, clinical evaluation included a medical history review and neuropsychological assessment. Based on this information, a multi-disciplinary clinical review panel determined each participant’s diagnosis and excluded participants that were ineligible. Participants were assigned a diagnosis of cognitively unimpaired (CU) if their performance was within 1.5 standard deviations of the published norms for their age group on neuropsychological assessment. A diagnosis of mild cognitive impairment (MCI) [21] or possible or probable Alzheimer’s dementia [22] were assigned according to internationally agreed criteria. This study was approved by the Austin Health Human Research Ethics Committee.

Between August 2018 and March 2020, all participants had a baseline Aβ-amyloid PET, tau PET, and 3-T structural MRI.

### Image acquisition

Aβ PET imaging involved the intravenous (IV) administration of 200 MBq (± 10%) [^18^F]NAV4694 with a 20-min acquisition time, commencing 50 min post-injection. Tau PET imaging involved the intravenous (IV) administration of 185 MBq (± 10%) of [^18^F]MK6240 with a 20-min acquisition time, commencing 90 min post-injection. All radiotracers were synthesized in-house at Austin Health, Melbourne, Australia. PET scans were acquired on one of two scanners: Philips TF64 PET/CT or a Siemens Biograph mCT. A low-dose CT was obtained for attenuation correction.

All participants underwent a structural MRI on a Siemens 3-T TIM Trio scanner (Siemens Medical Solutions) to obtain high-resolution T1-weighted anatomical magnetization-prepared rapid gradient echo (MPRAGE) sequences.

Aβ-amyloid PET scans were spatially normalized using CapAIBL [23], and the standard Centiloid (CL) method was applied for quantitation [24]. A Centiloid value > 25 was selected to determine a high Aβ scan [25–27]. [^18^F]MK6240 tau PET scans were spatially normalized using a CapAIBL PCA–based approach [28] and scaled using the cerebellar cortex as the reference region. [^18^F]MK6240 SUVR values were estimated in three composite volumes of interest, the mesial temporal (Me) comprising entorhinal cortex, hippocampus, parahippocampus, and amygdala; the temporoparietal (Te) composed of inferior and middle temporal, fusiform, supramarginal and angular gyri, posterior cingulate/precuneus, superior and inferior parietal, and lateral occipital; and the rest of neocortex (R), including dorsolateral and ventrolateral prefrontal, orbitofrontal cortex, gyrus rectus, superior temporal, and anterior cingulate [29]. We also estimated [18F]MK6240 SUVR in the entorhinal cortex, amygdala, hippocampus, and parahippocampus separately (Supplementary Materials). The threshold of tau positivity was defined as the 95%ile of the Aβ-negative (A-) participants in each composite VOI and mesial temporal regions. Around each threshold we also defined a peri-threshold zone comprised between the 90%ile and the 99%ile of the A- CU. Since previous studies reported tracer retention in the entorhinal cortex in low Aβ subjects [30, 31], and we had noted some discrepancy in individual cases where there was visual evidence of entorhinal T+ but the SUVR was below the threshold, the top 15% A- CU subjects with the highest entorhinal tau SUVR were visually classified as negative or positive in this region by consensus of three readers. We then used Youden’s index on visual classification to derive a SUVR threshold for entorhinal tau positivity.

We used two approaches to estimate the distribution of the prevalence of tau positive (T+) individuals along the Centiloid continuum. In the first approach, we used a simple histogram distribution of the prevalence of T+ subjects along a Centiloid *X*-axis, in bars of 20 CL. In the second approach, participants were ranked according to their Centiloid values, from the lowest (first participant) to the highest Centiloid (466th participant). A sliding window, *W*, captured blocks of 100 participants, rank-ordered by their Centiloid value. The window *W*_*i*_ includes participants *i*th to participant (*i +* 100)th. For each window, *W*_*i*_, we computed the prevalence (percentage) of tau-positive (T+) participants for each clinical group (CU, MCI, AD) and anchored it at (*CL*(*i*th) *+ CL* ((100 + *i*)th))/2*.*

While the variance is constant for each CL scale in the first approach, the window size depending of the CL distribution results in a variable bias. In the second approach, the bias is fixed for each Centiloid bar, but the variance depends on the Centiloid distribution. We chose to display both approaches to ensure our results are independent of any bias or variance.

## Results

The studied population were 60% CU, 20% mild cognitive impairment (MCI), and 20% possible or probable Alzheimer’s dementia (Table 1). The sex distribution was similar across groups, though the CU group had fewer males. Cognitively unimpaired subjects were older than MCI and AD. The AD had significantly more APOE4 carriers than CU. Seventeen percent of CU, 56% of MCI, and 80% of AD participants were Aβ positive (A+). The mean (SD) MMSE score in the cognitively unimpaired group was 28.6 (1.3) compared to 26.4 (2.3) in the MCI group and 22.8 (4.0) in the AD dementia group (Table 1).

**Table 1:** Demographics characteristics of the studied population

	CU	MCI	AD
Sample size	266	111	89
Sex, F (%)	151 (56)	52 (47)	38 (43)
Age	75.2 ± 5.5	73.1 ± 8.1**	71.0 ± 7.6***
Education (years)	14.0 ± 3.1	12.2 ± 3.1***	12.1 ± 3.1***
% APOE4	30.3	43.9	58.0***
Centiloid	15.8 ± 34.3	66.0 ± 63.0***	92.3 ± 59.1***^++^
% Aβ+	17.3	55.8	79.8
MMSE	28.6 ± 1.3	26.4 ± 2.3***	22.8 ± 4.0***^++^
CDR	0.08 ± 0.2	0.49 ± 0.1***	0.76 ± 0.5***
CDR SoB	0.1 ± 0.3	1.2 ± 0.7***	4.6 ± 2.3***^++^

***p value < 0.0005 compared to CU

^++^*p* value < 0.005 compared to MCI

The thresholds for tau positivity were 1.18 SUVR for Me, 1.24 SUVR for Te, and 1.08 SUVR for R. The peri-threshold ranges were 1.11 SUVR and 1.29 SUVR for Me, 1.19 and 1.33 SUVR for Te, and 1.0.4 and 1.17 SUVR for R. For the mesial regions, the tau thresholds [peri-thresholds] were 1.28 SUVR [no peri-threshold] for the entorhinal, 1.05 SUVR [0.98, 1.17] for the amygdala, 1.09 SUVR [1.03, 1.21] for the hippocampus, and 1.07 SUVR [1.01, 1.18] for the parahippocampus. All the thresholds for the mesial regions were of the same order except for the entorhinal cortex, which was higher. Compared to other mesial temporal regions, we noticed that the entorhinal cortex is in closer proximity to regions with high off-target binding, and the entorhinal signal may contain spillover from off-target binding.

**Fig. 1:**
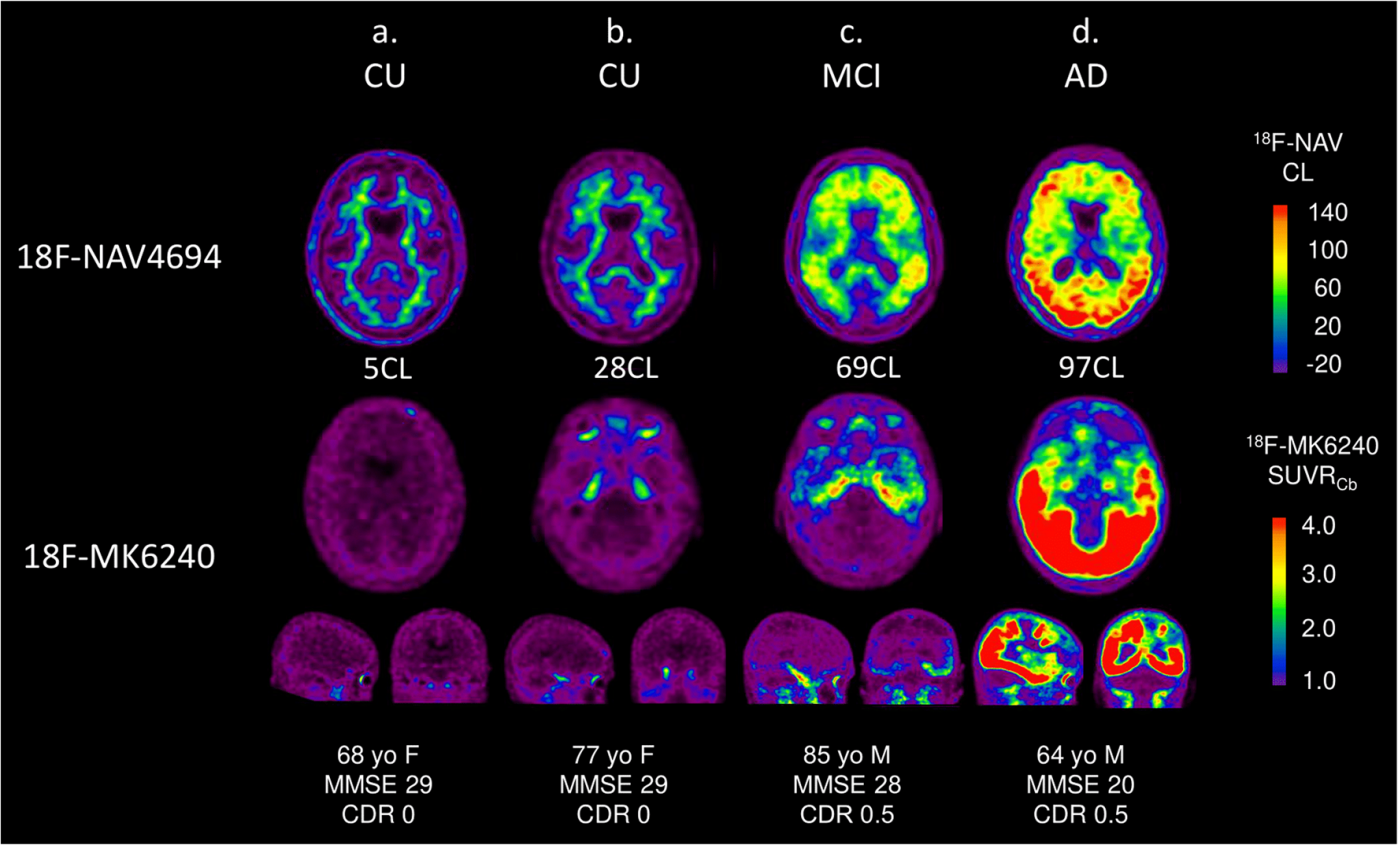
Representative axial, coronal, and sagittal Aβ and tau PET images across the spectrum of Centiloid values

Figure 1 displays the Aβ tau scans of several representative subjects along the Centiloid spectrum. On the left is a CU Aβ-negative individual with a [^18^F]MK6240 negative scan. On the second column, the CU individual has a Centiloid value just above the Aβ threshold, and there is noticeable [^18^F]MK6240 retention only in the MTL. The MCI participant has high Aβ burden (66 CL), with [^18^F]MK6240 retention in MTL and temporal neocortical areas. The last case is an AD patient, with a CL of 96 and widespread [^18^F]MK6240 retention in the neocortex.

**Fig. 2:**
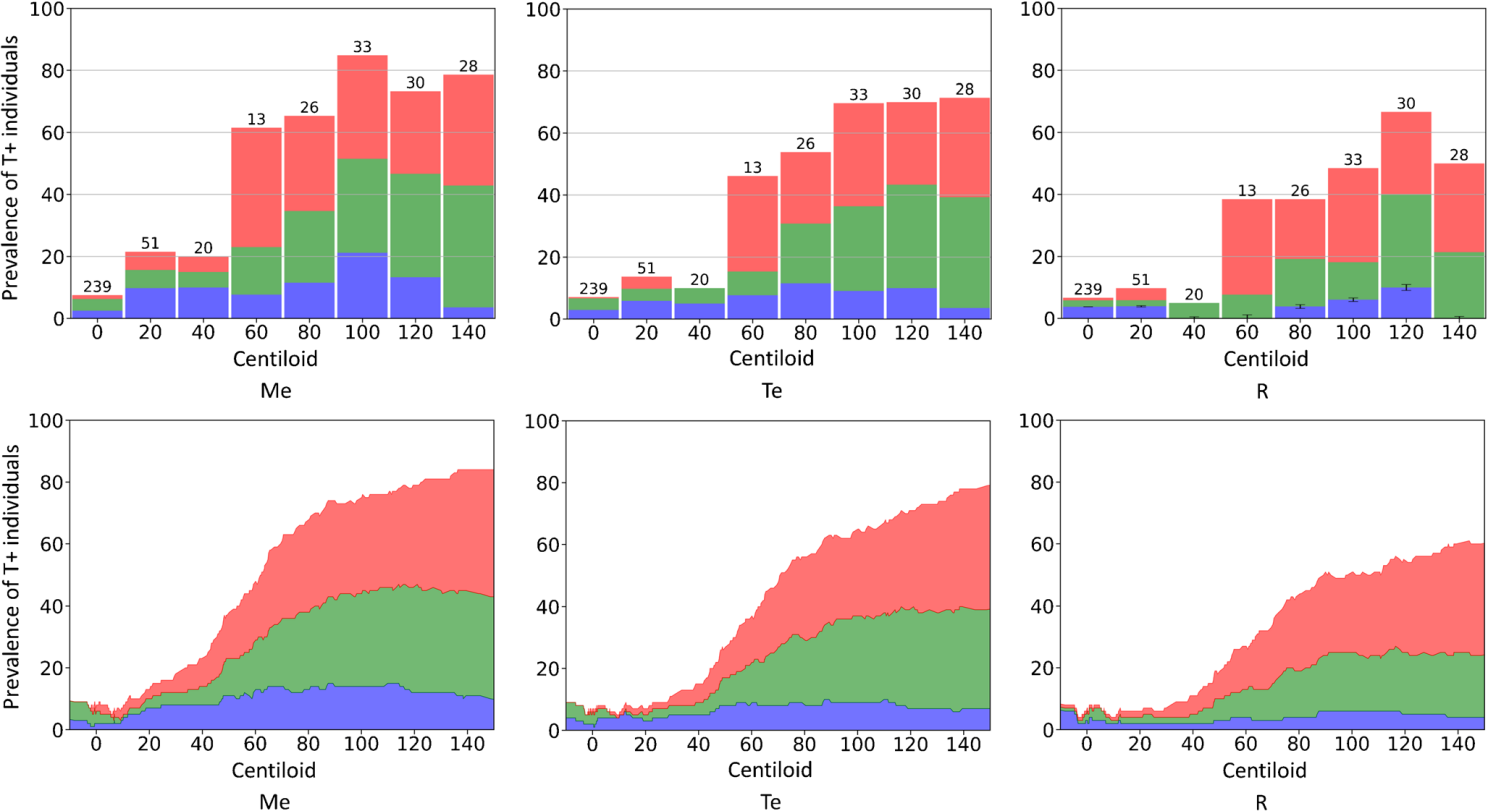
Histograms and stacked plot of the percentage of tau-positive subjects among the whole population versus Centiloid scores. Cognitive groups are colour-coded (CU: blue, MCI: green, AD: red)

The prevalence (%) of tau-positive (T+) individuals along the Centiloid continuum is shown in Fig. 2. Each clinical group was associated with a different colour (blue for CU, green for MCI, red for AD), and their prevalence was computed against the whole population. Hence, as an example, on the top left corner of Fig. 2, the bar at 60 CL indicates that 61% (8% CU, 15% MCI, and 38% AD) of the subjects with Centiloid between 50 and 70 CL had high tau in the medial temporal lobe (Me). Similar prevalence were observed when excluding subjects in the peri-threshold region (Online Resource 1). Stacked plot and histograms showed similar results. There was no association between prevalence of T+ individuals and CL among individuals with CL < 10. Only 7.6% (CU: 2.0%, MCI: 3.8%, AD: 1.8%) of the individuals with a CL < 10 had high tau in Me (T_ME_+), 6.9% (CU: 3.0%, MCI: 3.2%, AD: 0.7%) also had a high tau in Te (T_Te_+), and 6.4% (CU: 3.0%, MCI: 2.5%, AD: 0.9%) in R were T_R_+. These results reflect the use of the 95th percentile of A- CU to define the threshold so suggest no increase in tau in any individual with < 10 CL of Aβ. The prevalence of T+ among subjects with CL between 10 and 40 was linearly associated (*p* < 10^¬5^) with CL scale in the three composite ROIs. For each Centiloid unit, the prevalence of T+ subjects increased by 0.48% in Me (CI = [0.46%, 0.50%]), by 0.24% in Te (CI = [0.21%, 0.27%]) and by 0.15% in R (CI = [0.12%, 0.17%]) reaching 23%, 15%, and 11% respectively of the whole population at 40 CL. The same linear slopes were observed when removing subjects in the peri-threshold zone. The prevalence of high-tau subjects in the 40 CL to 70 CL range was also linearly associated (*p* < 10^−5^) with CL but with a steeper slope 1.26%/CL (CI = [1.22%, 1.30%]) in Me, 1.16%/CL in Te (CI = [1.12%, 1.19%]), and 0.83%/CL in R (CI = [0.80%, 0.86%]). When excluding the subjects in the peri-threshold zone, the slopes were higher (1.6%/CL, 1.4%/CL, and 1.0%/CL, respectively). At 70 CL, 64% of the population were T+ in Me, 51% were T+ in Te, and 37% in R. After 70 CL, the prevalence of high-tau subjects continued to increase but not linearly in Me, reaching 86% of the population at 150 CL (81% in Te and 62% in R, respectively). The same prevalence was found when excluding subjects in the peri-threshold zone. The main difference between Me and Te VOIs was observed before 40 CL, where the slope of the prevalence of Me T+ was nearly double that of the Te region without being significant. T+ CU were mostly A+ subjects (i.e. > 25 CL).

Supplementary Fig. 2 shows the prevalence of tau-positive (T+) individuals in the entorhinal, amygdala, hippocampus, and parahippocampus regions along the Centiloid continuum. The prevalence of T+ CU in the entorhinal was the highest (15% of the CU with CL < 25). At 40 CL, 40% (CU 30%, MCI 10%, AD 10%) of the individuals were T+ in the entorhinal and at 60 CL, almost 80% (CU 15%, MCI 23%, and AD 38%). Prevalence remained stable above 60 CL. The graphs of the T+ prevalence in the amygdala, hippocampus, and parahippocampus followed the same trend as in the entorhinal but with lower prevalence; among these three mesial regions, the amygdala had the highest prevalence followed by the hippocampus and the parahippocampus.

**Fig. 3:**
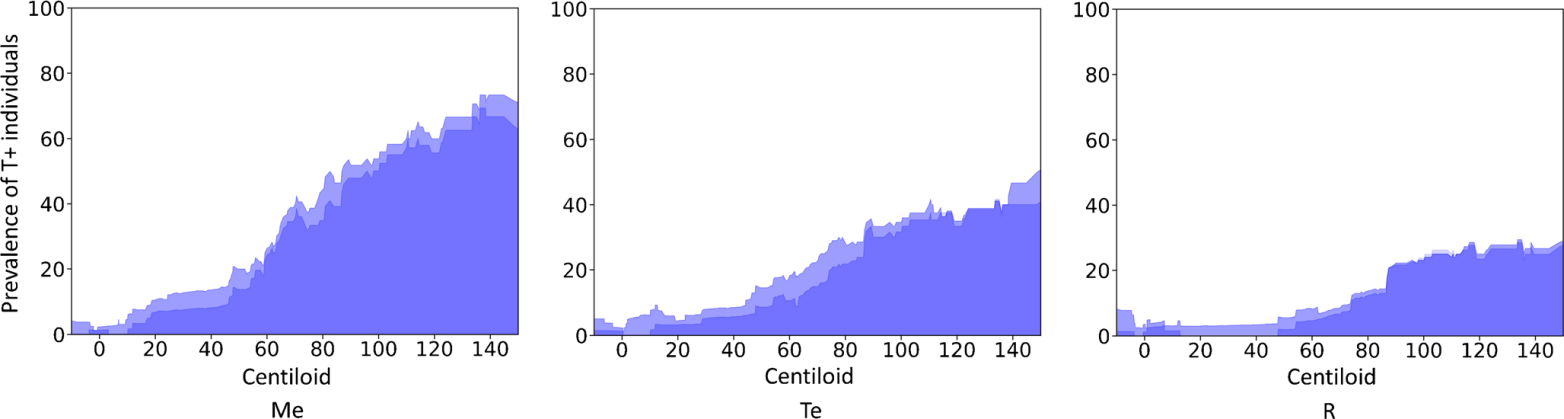
Prevalence (percentage) of high-tau (T+) CU subjects among the cognitively unimpaired subjects

Figure 3 illustrates the prevalence of T+ CU among CU individuals (the dark blue curve excludes individuals in the tau peri-threshold zone). There were very few T+ CU subjects in Me (2.5%) and Te and R (4.0%) among subjects with a Centiloid value lower than 10 CL. In Me, there was a step-like increase of the prevalence of T+ CU individuals between 10 and 20 CL (15%) and then a slow increase up to 40 CL (17%). The mean prevalence of T+ CU between 10 and 40 CL was 10% in Te and 5% in R; however, half of them were in the Te tau peri-threshold range while all were below the R upper peri-threshold level. Above 50 CL, the prevalence of high-tau subjects drastically increased to reach 65% of the CU at 150 CL in Me, while the increase was moderate in the cortical regions at 50% in Te and 28% in R at 150 CL. The results for the entorhinal cortex, amygdala, hippocampus, and parahippocampus are shown in Supplementary Fig. 3. The step-like increase observed between 10 and 20 CL in Me is also observed in all mesial regions. As discussed above, the prevalence of T+ CU was the highest in the entorhinal cortex for all Centiloid values (reaching 40% of the CU at 30CL and 80% at 100 CL) followed by the amygdala, the hippocampus, and finally the parahippocampus.

**Fig. 4:**
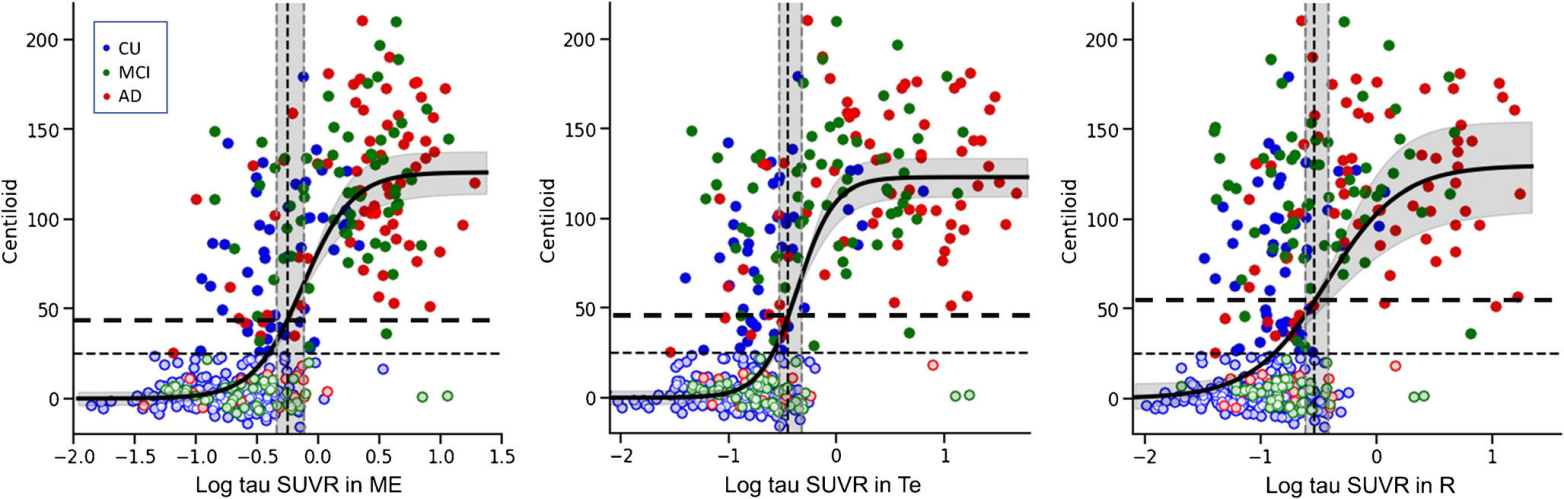
Scatter plot of Centiloid versus the log tau SUVR in the Me (left) and Te (middle) and R (right) ROI in 3 different colour-coded clinical diagnostic groups, with light colours used to identify A- subjects. Thresholds are displayed in fine dash vertical and horizontal lines*.* Peri-thresholds are in grey shadow and a sigmoid curve has been fitted to the whole population. The thick dash horizontal line shows the CL value at which tau SUVR reaches the threshold for elevated tau.

Figure 4 shows the scatter plot of the Centiloid versus the log tau SUVR in Me, Te, and R. We used a log scale for the tau SUVR to stretch its distribution in the lower SUVR range and compress it in the high SUVR range. The Spearman’s rank correlations were *p* = 0.62 (Me), *p* = 0.51 (Te), and *p* = 0.42 (R) (*p* value < 10^–20^) and the best fit was obtained with a sigmoid curve. The lower asymptote of the sigmoid was 0 CL, corresponding to the mean Centiloid of the lowest tau SUVR subjects, while the upper asymptote was ~ 125 CL, representing the average amount of amyloid in subjects with the highest tau tracer retention. This upper mark is of the same scale as the mean AD CL value (100 CL) even though the CL of subjects with high-tau tracer retention ranged between 50 and 200 CL. The sigmoid crossed the 25 CL threshold for Aβ PET at tau PET levels at 1.06 SUVR_ME_, 1.15 SUVR_Te_, and 0.92 SUVR_R_, and were still below the tau PET threshold. The threshold for Me tau PET was crossed at 43.9 CL (peri-threshold [32.8 CL, 64.7 CL] and the Te tau threshold crossed at 44.6 CL (peri-threshold [32.6 CL, 67.2 CL]) and the R tau threshold crossed at 53.9 CL [64.3 CL, 66.8 CL]). Similar plots for the mesial regions are also presented in Supplementary Fig. 4. The sigmoid crossed the Aβ PET threshold of 25 CL at tau PET levels of 1.28 SUVR_enthorinal_, 0.93 SUVR_amygdala_, 0.96 SUVR_hippocampus_, and 0.93 SUVR_parahippocampus_, respectively. The threshold for the entorhinal tau PET was crossed at 25 CL while the tau threshold crossed at 40 CL for the amygdala, 50 CL for the hippocampus, and 57 CL for the parahippocampus.

**Fig. 5:**
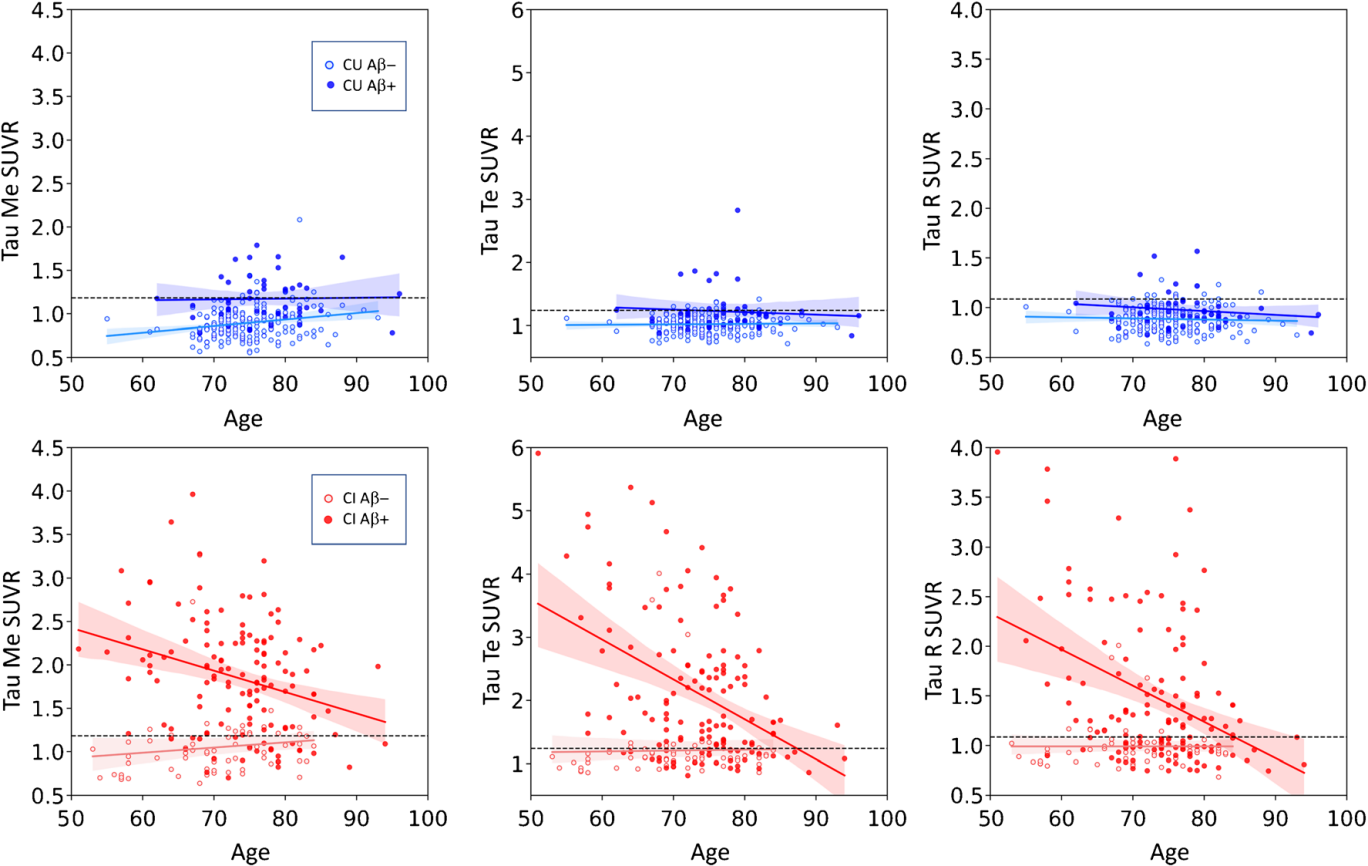
Scatter plot of tau SUVR in the Me (left) and Te (middle) and R (right) ROI versus age at tau scan in 2 different colour-coded clinical diagnostic groups (cognitively unimpaired (CU) and cognitively impaired (CI)), with light colours used to identify A- subjects

Figure 5 shows the association between tau SUVR and age. In this graph, we have combined AD and MCI in cognitively impaired (CI) subjects. We found a positive association between CU A- subjects and age (*r* > 0.2, *p* < 0.0001) while we found a negative association between CI A+ in the three regions of interest (*r* < − 0.3, *p* < 0.0005). No correlation was found in the other groups or regions.

## Discussion

Several studies have shown evidence that high cortical Aβ triggers the spread of tau outside the MTL region (Price and Morris 1999; Scholl 2016). The aim of this study was to estimate at which CL level abnormal tau can be detected on PET imaging in medial temporal and neocortical regions.

The Me and the Te masks provided similar prevalence results. The main differences were observed in the low and moderate CL values (< 40 CL), where the prevalence of the T+ subjects in Me were higher than their prevalence in the Te, especially for the cognitively unimpaired group. This is in agreement with previous neuropathological reports showing tau accumulation in MTL before cortical accumulation of Aβ [32]. We thus found that the increase in T+ prevalence was small and linear in Me and in Te between 10 and 40 CL (0.48%/CL in Me and 0.24%/CL in Te) and was in part driven by A-T+ subjects. A large proportion of these A-T+ subjects had a regional tau SUVR in the tau peri-threshold zone. In mesial temporal regions (entorhinal cortex, amygdala, hippocampus, and parahippocampus), we found the prevalence of T+ subjects was much higher in the entorhinal cortex than in the other mesial temporal regions, suggesting that some individuals may have detectable tau only in the entorhinal cortex, even in the absence of Aβ. The sequence of the highest prevalence (entorhinal, amygdala, hippocampus, and parahippocampus) may relate to the sequential spread of tau in the mesial temporal cortex that will be clarified with longitudinal analysis.

At 40 CL, 23% of the subjects had a high-tau SUVR in Me and 15% in the Te region; however, most of these T+ results were close to the tau thresholds. Around the 40 CL mark, there was a steep increase in the prevalence of T+ subjects in all cognitive groups, including CU subjects. This finding is consistent with the 50 CL threshold we found in our post-mortem study for the presence of Alzheimer’s Disease Neuropathic Change (ADNC) sufficient to meet the criteria for Alzheimer’s disease [25]. It is also consistent with our previous finding that an Aβ level of greater than 50 CL is required to see a substantial increase in the risk of developing mild cognitive decline over 5-year follow-up in cognitively unimpaired participants in the AIBL study [33]. The increase of prevalence in our model shows that in a few individuals, tau deposits are detectable when sparse Aβ-amyloid is present, while for the vast majority, tau scans are only positive when amyloidosis is well-established.

As previously reported in the literature with [^18^F]Flortaucipir [34–36], we found a positive association between tau SUVR and age in the mesial temporal of the CU and a strong negative association in the cognitively impaired subjects in the three regions of interest. The current interpretation of this peculiarity is that older people are more likely to have comorbidities than younger persons. These comorbidities are also associated with cognition, and therefore, a lower level of tau pathology is necessary in older ages to reach similar levels of cognitive impairment. This study has some limitations. First, in vivo PET imaging is limited by the detection sensitivity of the technique (goodness of the measure), by the Aβ and tau tracers’ kinetics, affinity and stoichiometry, and by the density of available binding sites of each target. These findings are specific to the two PET tracers, specifically [^18^F]NAV4694 and [^18^F]MK6240 and findings can differ from other tracers. As a new tracer, there is not yet autopsy validation of [^18^F]MK6240 PET so, though it performs well compared to other tau tracers, the sensitivity for in vivo detection of low tau levels or low Braak stages of tau deposition is uncertain. Also, results are determined by the threshold used to define tau positive (T+). A more liberal or conservative threshold for T+ may yield slightly different results. In this paper, we opted for large sliding windows (Fig. 2) to smooth the noise and only highlight the general trend. Narrower windows provided a more detailed picture of the relationship between Centiloid and tau; however, noise was very high and obscured the general trend. Larger cohorts would allow for a more detailed relationship. Finally, our study is limited due to its cross-sectional nature and future longitudinal analysis is needed to confirm our findings.

## Summary

This study supports several lines of investigation that suggest neocortical tau deposition is rarely seen with an Aβ level below 40 CL and above this Aβ level the prevalence of abnormal tau deposition in all brain areas accelerates.

## Supplementary information

ESM 1 (DOCX 1226 kb).

## Data Availability

The datasets used and/or analysed during the current study are available from the corresponding author on reasonable request.
