# Supplementary material for: Relationship between amyloid and tau levels and its impact on tau spreading
Source: Eur J Nucl Med Mol Imaging. 2021 Jan 26;48(7):2225–32. doi: 10.1007/s00259-021-05191-9 (PMC8175299; doi:10.1007/s00259-021-05191-9)
Supplement: Supplementary file 1 — (DOCX 1226 kb). [file 259_2021_5191_MOESM1_ESM.docx]

**Supplementary material**


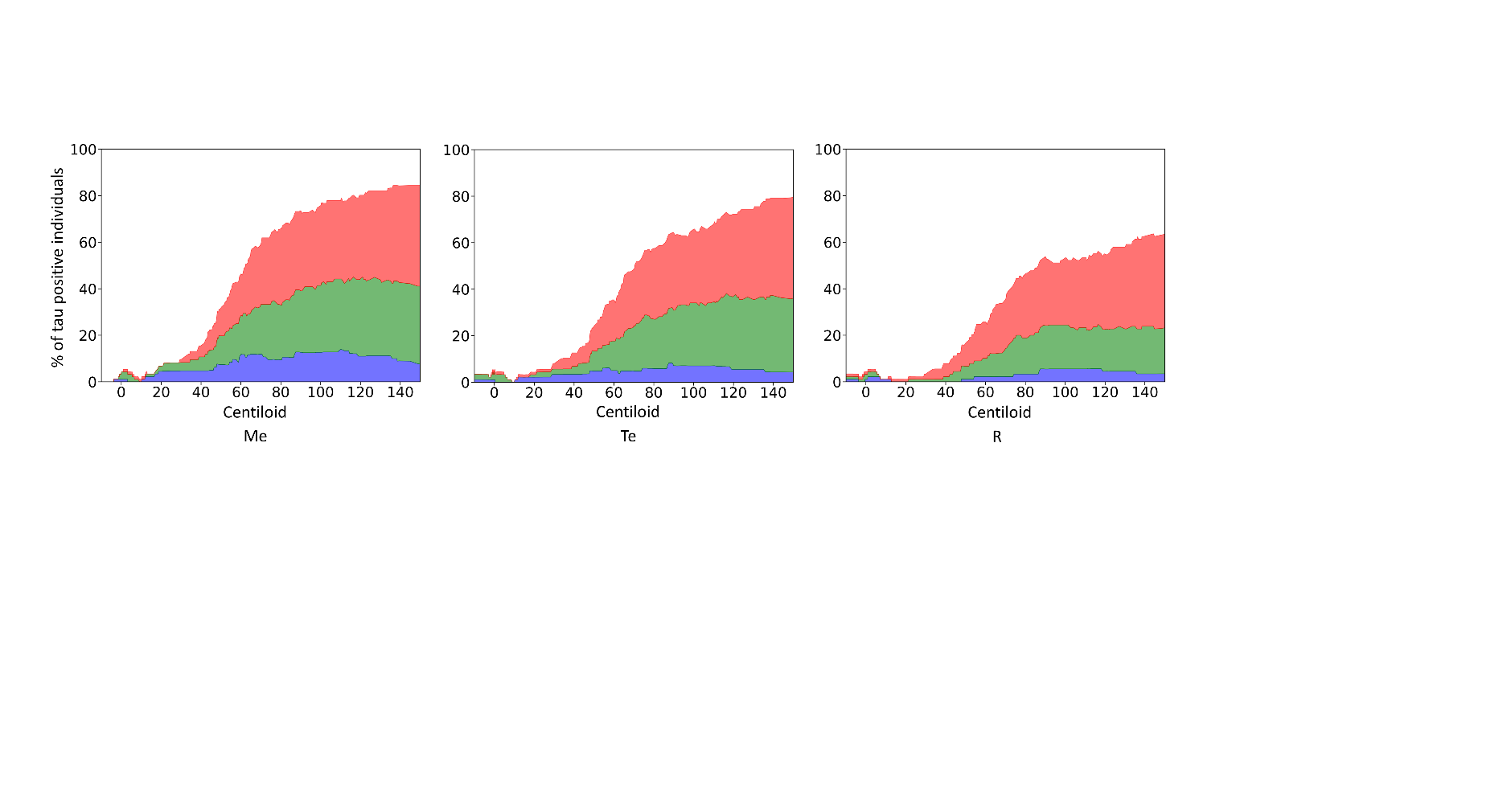


**Online Resource 1** Prevalence of tau positive subjects as a function of centiloid excluding subjects in the peri-threshold.


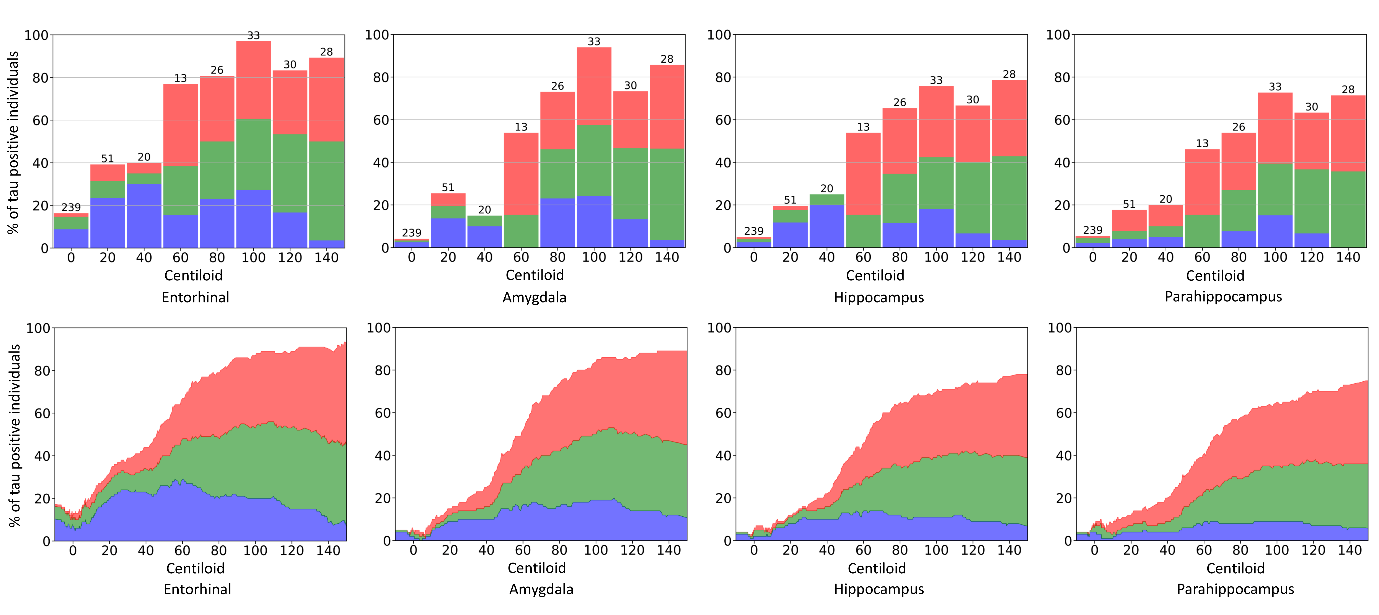


**Online Resource 2** Prevalence of tau positive subjects as a function of centiloid in the 4 subregions of Me.


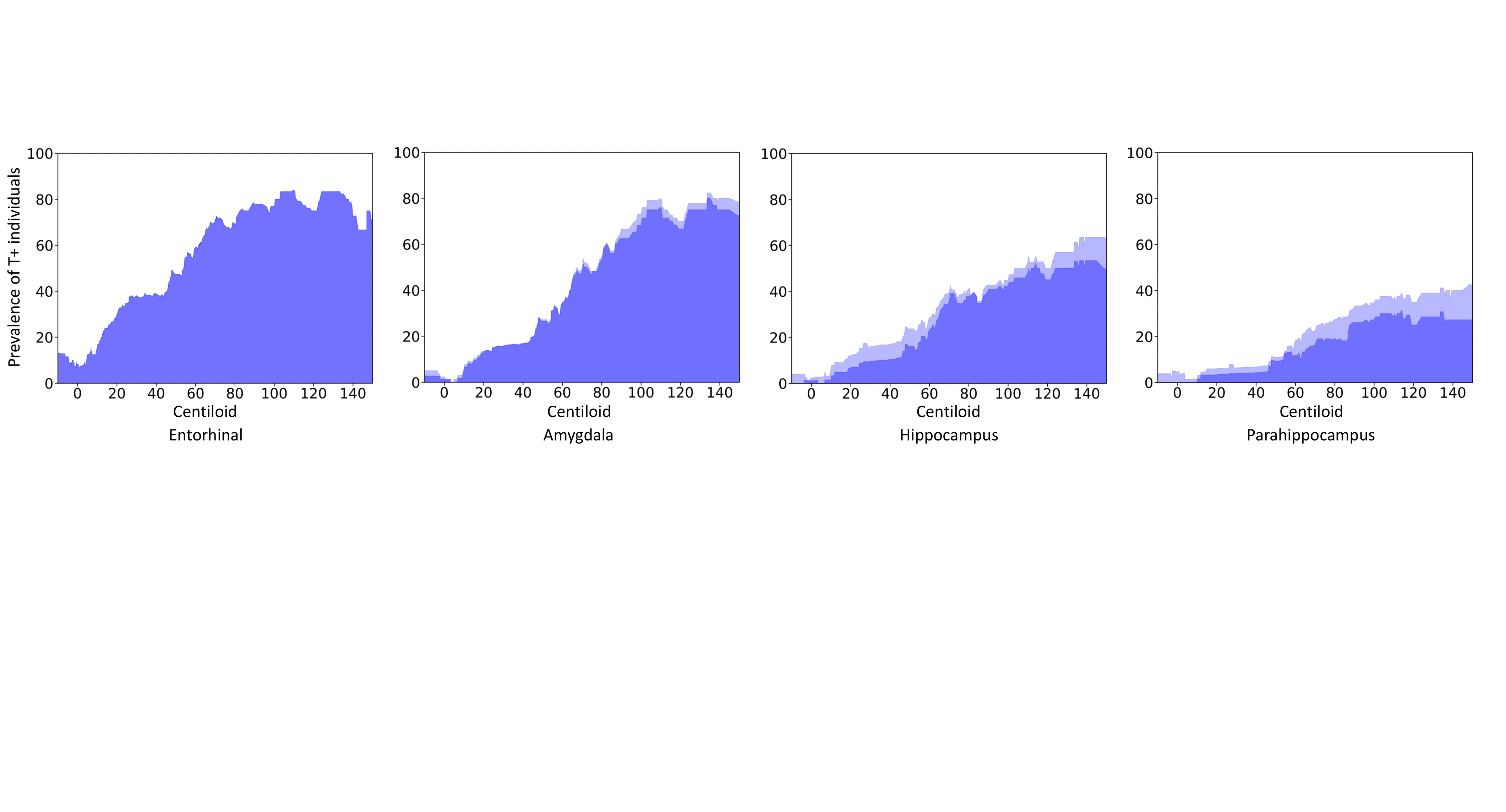


**Online Resource 3** Prevalence (percentage) of high tau CU subjects among the cognitively unimpaired in the 4 subregions of Me. No peri-threshold were available for the entorhinal cortex


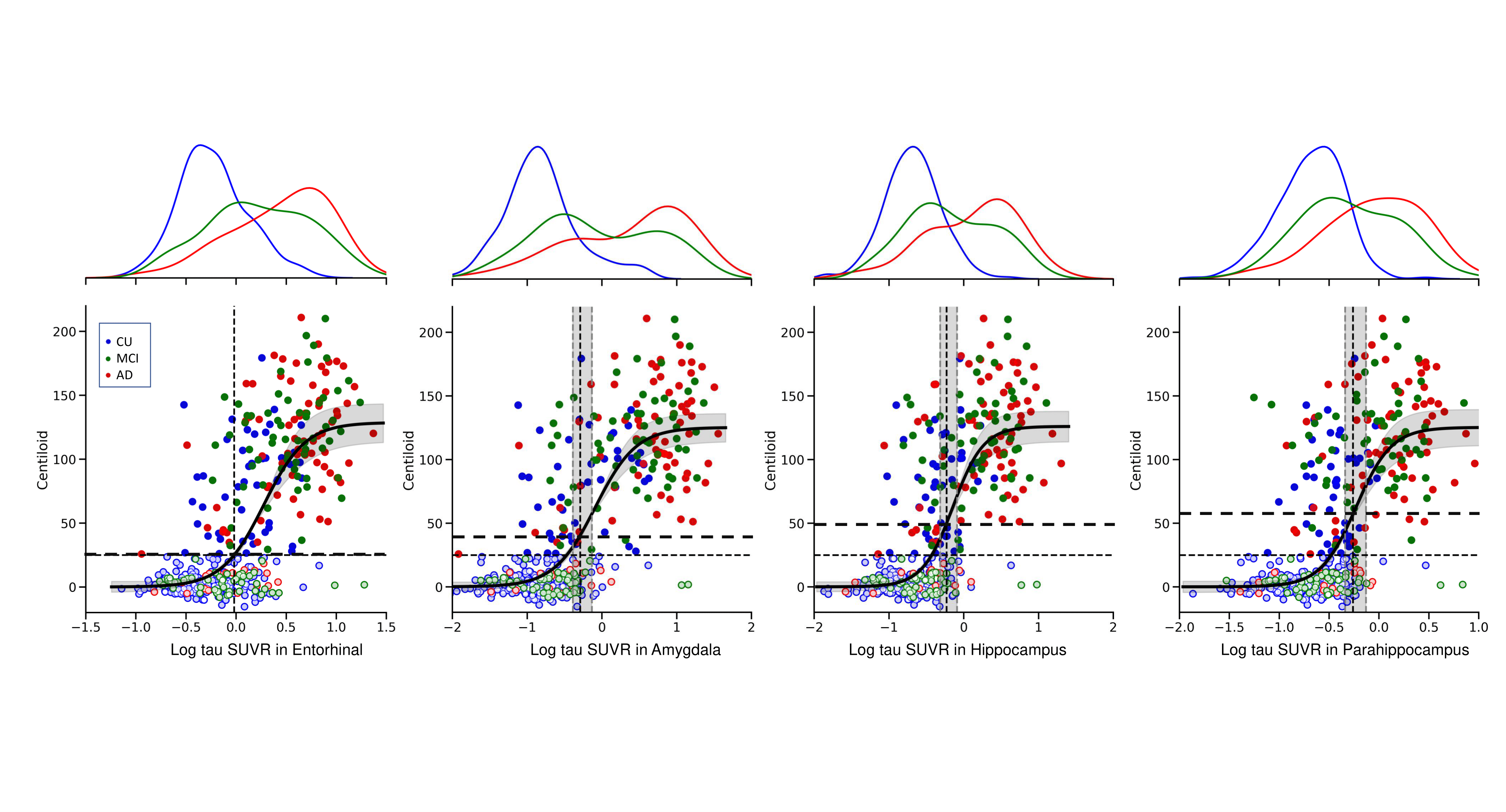


**Online Resource 4** From left to right, scatter plots of Centiloid versus the log tau SUVR in the entorhinal cortex (left), amygdala, hippocampus and parahippocamus ROI in 3 different color-coded clinical diagnostic groups, with light colours used to identify A- subjects. Thresholds are displayed in fine dash vertical and horizontal lines. Peri-thresholds are in grey shadow and a sigmoid curve has been fitted to the whole population. The thick dash horizontal line shows the CL value at which tau SUVR reaches the threshold for elevated tau. No peri-threshold were available for the entorhinal cortex
